# Selective admission into stroke unit and patient outcomes: a tale of four cities

**DOI:** 10.1186/2191-1991-4-1

**Published:** 2014-01-09

**Authors:** Farasat AS Bokhari, Ian Wellwood, Anthony G Rudd, Peter Langhorne, Martin S Dennis, Charles DA Wolfe

**Affiliations:** 1 School of Economics and ESRC Centre for Competition Policy, University of East Anglia, Norwich, UK; 2 Department of Primary Care and Public Health Sciences, Division of Health and Social Care Research, King’s College London, London, UK and NIHR Biomedical Research Centre, Guy’s & St Thomas’ NHS Foundation Trust, King’s College London, London, UK; 3 Academic Section of Geriatric Medicine, University of Glasgow, Glasgow, UK; 4 Division of Clinical Neurosciences, Western General Hospital, Edinburgh, UK

**Keywords:** Stroke unit care, Selection bias, Outcomes, Propensity score matching

## Abstract

Care of stroke patients costs considerably more in specialized stroke units (SU) compared to care in general medical wards (GMW) but the technology may be cost effective if it leads to significantly improved outcomes. While randomized control trials show better outcomes for stroke patients admitted to SU, observational studies report mixed findings. In this paper we use individual level data from first-ever stroke patients in four European cities and find evidence of selection by the initial severity of stroke into SU in some cities. In these cases, the impact of admission to SU on outcomes is overestimated by multivariate logit models even after controlling for case-mix. However, when the imbalance in patient characteristics and severity of stroke by admission to SU and GMW is adjusted using propensity score methods, the differences in outcomes are no longer statistically significant in most cases. Our analysis explains why earlier studies using observational data have found mixed results on the benefits of admission to SU.

## Background

European health care systems have significant differences among them but a common feature is to typically rely on a coordinated or centrally planned decision about the adoption of new technologies and services. In that respect, cost-effectiveness analysis plays an important role in this decision. For instance, in the U.K., the National Institutes of Clinical Excellence (NICE) recommends that a new technology be adopted if the cost per quality adjust life year (QALY) is £30,000 or less. However, analysis based on observational data, as opposed to randomized control trials, may reach different conclusions about the relative effectiveness (or cost effectiveness) of a new service. In part this may be due to selective assignment of patients to new technologies or services relative to the standard care in different centers.

In this paper we use observational data on patient outcomes from four European cities on stroke patients who are exposed to an expensive technology – admission to a specialized stroke unit ward (SU) or general medical wards (GMW), and demonstrate the importance of correcting for selection bias in measuring differences in outcomes (which in turn are used in calculations of incremental cost effectiveness ratios). We find evidence of overt selection into SU in some cities, and, in these cases, find that the impact of admission to SU on outcomes is overestimated by standard multivariate logit models even after controlling for case-mix. Using propensity score methods to control for selection, we do not find evidence of differential outcomes by admission to SU or other wards. Our analysis explains why earlier studies using observational data have found mixed results on the benefits of admission to SU.

Selection bias is said to occur when patients with specific characteristics are systematically assigned to treatment A or B and these characteristics independently also influence the outcome. Reasons for such selections may stem from a *de facto* policy of admitting patients with given risk factors into the more intensive treatment group [1]. Alternatively, physicians’ aversion to risk may lead to higher risk patients receiving more conservative care [2,3]. For instance, in interventions for cardiovascular disease, older and high risk patients with chronic kidney disease are often treated more conservatively despite the fact that these higher risk patients may receive greater benefit from coronary angiography after acute myocardial infarction [2]. Similarly, Dranove et al. showed that publication of 'report cards’ on health care providers in New York and Pennsylvania led to selection behavior where both doctors and hospitals preferred to treat healthier patients for coronary artery bypass graft (CABG) surgery, which in turn decreased patient and social welfare [3]. In observational studies, *overt* selection bias occurs when there is an imbalance in the characteristics of patients assigned to the treatment or control groups and these characteristics also influence the outcome [4,5].

In the context of admission to stroke unit, this can happen if patients with certain characteristics, e.g., type of stroke, severity, age, pre-existing conditions, are either systematically admitted to stroke units or admitted to other wards since these characteristics also independently influence patient outcome. The bias may be favorable in one center (healthier stroke patients admitted to stroke unit in one city), unfavorable in another (triaging those with worse case-mix into stroke units) and yet may not be present in other cases. Similarly, hidden or *covert* bias can occur if patients in treatment or control groups differ in unobservables and these differences also influence outcomes.

Regardless of the reasons for selection bias – gaming, triaging, defacto policies or constraints of the health care system – estimation methods that do not explicitly account for these differences lead to biased estimates of the treatment effect [6-8]. Traditional case-mix adjustments via multivariate regression based methods cannot completely account for the selection bias. In the presence of overt bias, propensity score methods have been proposed as alternatives for estimating treatment effect. Under this method, one first computes the probability that a patient is assigned to treatment or control group conditional on the observed characteristics, and then conditional on this score, compares outcomes between the two groups [5,9,10]. Rosenbaum and Rubin showed that under the assumption of strongly ignorable treatment assignment, given the observed covariates, selection bias generated by the differences in observed covariate values between the two groups can be removed [4]. Nonetheless, in a systematic review of 43 studies, Shah et al. concluded that regressions and propensity score methods gave similar results, but also found that odds or hazard ratios computed using propensity score methods were, on average, 6.4% of the times closer to one compared to traditional regression based methods, and that the statistical significance across the two methods differed in only about 10% of the cases [11]. Similarly, Stürmer et al. report that in only 9 of 69 studies, the effect estimate differed by more than 20% across the two methods [12].

In the present study, we compare outcomes for stroke patients admitted to specialist stroke units (SU) and other general medical wards (GMW) from four different cities in Europe. We pay particular attention to the issue of selection bias and explicitly model the likelihood of a patient being admitted to SU given their case-mix, and importantly, the measured severity of stroke prior to admission to SU or GMW. We use both the regression based and propensity score based methods and highlight the differences in estimated treatment effects across these methods. Pooling observations from multiple centers creates the added difficulty of appropriately accounting for heterogeneity of services in SU across centers. Thus, to account for heterogeneity of services in SU across the four centers, as well as to allow for possibly different forms of selection bias (favorable, unfavorable or none), we analyze data from each of the four cities separately. This then allows us to detect selection and account for it when comparing outcomes in SU versus GMW within each city rather than confound the results by comparing across non-homogeneous SU centers. For comparison, we also show results from pooling all observations across centers and ignoring the underlying heterogeneity across centers.

## Methods

### Source, sample and variables

Data for this study are drawn from the European Register of Stroke Collaborators (EROS) database and is a collection of population based registers established in six European cities including Dijon (France), Sesto Fiorentino (Italy), Kaunas (Lithuania), Warsaw (Poland), Mahon, Menorca (Spain) and London (UK). The database has been described in detail elsewhere and we restrict our description to the selected sample for this study [13,14]. The data collection took place between 2004 and 2006 and provides detailed information on 2,127 first-ever stroke patients from an estimated 2005 source population of 1,087,048. The database includes patient information on demography, comorbidities, living conditions prior to stroke, severity and type of stroke, whether the patient was admitted to a hospital, type of hospital ward admitted to, length of stay, and types and counts of services and therapies provided to the patient. Patients were followed for up to one year in all centers (with some centers following patients for two years) and consequently mortality outcome is known.

The three main outcome measures were mortality at one month, three months and one year and are analyzed by whether a patient is admitted to SU or GMW. Two centers, Warsaw and Menorca, were omitted from this analysis due to lack of variation in type of admission (SU or GMW). In Warsaw, all but four patients were admitted to SU, while in Menorca only one patient was admitted to SU. Within each center we restricted our attention to patients who either suffered cerebral infarction (CI) or primary intracerebral haemorrhage (PICH), as these patients would be typically eligible for SU care. Among the remaining four centers, 35 patients were not admitted to any hospital, the type of ward is not known for four patients, 49 patients had a subarachnoid haemorrhage (SAH), and 42 had an unknown or unclassified type of stroke. This led to an exclusion of 128 observations (there was some overlap) giving a potential sample of 1,693 first-ever stroke patients with either first-time stroke as CI or PICH. The final sample consisted of 1,492 observations, where the additional loss of observations was because complete information on case-mix variables (described below) was not available.

Severity of stroke at initial diagnosis is measured using two alternative scales, the Glasgow Coma Scale (GCS) and the National Institutes of Health Stroke Scale (NIHSS) [14]. Glasgow Coma Scale is a neurological scale that measures the conscious state of a patient and is used for both initial as well as subsequent assessment. The scale consists of a sum of numerical scores assigned to three components that test for eye, verbal and motor responses (assigned from 1–4 or 1–6 for different components) where the lowest possible value of 3 for GCS means deep coma or death while the highest value of 15 implies a fully awake person. Our primary analysis uses GCS as a measure of severity of stroke because it is available in our data set for all patients (at the time of admission) whereas the NIHSS (an alternative scale) is not recorded for about 25% of our working sample and hence serves as a secondary measure for robustness checks. In our sensitivity analysis we also used alternative forms of the GCS variable. For instance, rather than use the value of GCS, we used individual components of GCS by eye movement, motor movement and verbal component scores, and also repeated the analysis treating GCS as a categorical variable (low, medium and high). Several other variants of the model were also estimated and results from those are discussed in the Robustness section.

Other relevant controls include age, age square, gender, interaction of age and gender, type of stroke (CI or PICH), swallowing test, ability to lift both arms, ability to walk, pre-stroke comorbidities (Atrial Fibrillation (AF), Myocardial Infarction (MI), Diabetes Mellitus (DM), Hypertension (HT)), whether the patient was independent or needed assistance in the two weeks prior to stroke and living conditions prior to stroke (home alone, home with others, supportive living/nursing home, long term hospital care, or other/unknown). This last variable, living conditions prior to stroke, is not coded uniformly across centers and hence we accordingly use only categories that are available. For instance, in the case of London and Dijon, patients are coded according to all five categories but in the case of Florence all patients in the sample are either coded as home alone or home with others. Data are summarized in Table 1.

**Table 1:** Mean value of outcome and GCS scores by admission to SU and GMW

	**London**	**Kaunas**	**Dijon**	**Florence**	**Combined**
	**For all patients**				
Number of patients	365	699	335	93	1492
Mortality (30 days)	17%	15%	5%	13%	13%
Mortality (90 days)	21%	19%	8%	20%	17%
Mortality (365 days)	28%	25%	15%	24%	24%
Glasgow Coma Score (GCS)	12.79	13.72	14.51	13.43	13.65
	**For patients admitted to SU**				
Number of patients	292	77	132	33	534
Mortality (30 days)	12%	44%	4%	15%	15%
Mortality (90 days)	18%	56%	5%	24%	20%
Mortality (365 days)	25%	68%	10%	27%	27%
Glasgow Coma Score (GCS)	13.19	11.47	14.64	13.15	13.30
	**For patients admitted to GMW**				
Number of patients	73	622	203	60	958
Mortality (30 days)	36%	11%	5%	12%	12%
Mortality (90 days)	37%	14%	10%	18%	15%
Mortality (365 days)	43%	20%	18%	22%	21%
Glasgow Coma Score (GCS)	11.18	14.00	14.42	13.58	13.85

Abbreviations: Glasgow Coma Scale (GCS); Stroke Unit (SU); General Medical Ward (GMW).

### Analysis – multivariate logits

For each of the three outcome measures (mortality at 30, 90 and 365 days), multivariate logit models were estimated to compute the odds ratio of mortality for admission to SU relative to GMW by center, controlling for severity of stroke and other relevant risk factors (i.e., demographic and case-mix adjustments). Thus for each of the four centers and three outcomes (*c* = 1,2,3,4 and *o* = 1,2,3) we estimated,

(1) $$\text{Pr}\left(Y_{i}^{\text{co}}=1\right)=\Lambda \left(\beta_{1}^{\text{co}}SU_{i}+\beta_{2}^{\text{co}}{\text{SS}}_{i}+\beta_{3}^{\text{co}}X_{i}\right)$$

where *SS*_*i*_ is a measure of severity of stroke at initial diagnosis (GCS or NIHSS), *X*_*i*_ is a vector of other relevant risk factors (i.e., demographic and case-mix adjustments listed earlier) for the *ith* patient and the primary coefficient of interest is *β*_1_ which provides the odds ratio for admission to SU (by center *c* and outcome *o*).

Additionally, for each center, we also estimated odds ratio for admission to stroke unit as a function of severity of stroke. In this second model, the primary interest is in detecting whether the severity of stroke (GCS or NIHSS) predicts if a patient is admitted to SU or GMW, after controlling for other observable characteristics of the patients (case-mix, demographic variables, and other pre-stroke conditions). Thus for each of the four centers, we estimated

(2) $$\text{Pr}\left({\text{SU}}_{i}^{c}=1\right)=\Lambda \left(\theta_{1}^{c}{\text{SS}}_{i}+\theta_{2}^{c}Z_{i}\right)$$

where *SS*_*i*_ is the measure of severity of stroke at initial diagnosis, *Z*_*i*_ is the vector of other controls, and *θ*_1_ is the coefficient of interest which provides odds of admission to SU as a function of severity of stroke. Results from multivariate logits are summarized in Table 2.

**Table 2:** **Selected odds ratios (OR) and confidence intervals**^**1**^

**Outcome**	**London**	**Kaunas**	**Dijon**	**Florence**	**Combined**
**Panel A**	**Unadjusted odds ratios for outcomes by admission to SU**				
Mortality (30 days)	0.25 [0.14,0.45]	6.34 [3.79,10.6]	0.69 [0.23,2.03]	1.35 [0.39,4.65]	1.29 [0.95,1.77]
Mortality (90 days)	0.36 [0.21,0.63]	7.78 [4.70,12.9]	0.44 [0.17,1.12]	1.43 [0.51,3.99]	1.42 [1.08,1.87]
Mortality (365 days)	0.44 [0.26,0.76]	8.35 [4.99,14.0]	0.51 [0.26,1.00]	1.36 [0.51,3.62]	1.39 [1.09,1.78]
**Panel B**	**Adjusted odds ratios for outcomes by admission to SU**^**2**^				
Mortality (30 days)	0.12 [0.03,0.46]	1.40 [0.69,2.84]	0.93 [0.17,4.91]	3.11 [0.16,59.1]	0.85 [0.53,1.36]
Mortality (90 days)	0.35 [0.11,1.11]	1.98 [0.99,3.97]	0.58 [0.17,2.00]	3.89 [0.30,50.7]	1.18 [0.79,1.78]
Mortality (365 days)	0.55 [0.23,1.29]	2.09 [1.07,4.06]	0.80 [0.34,1.90]	1.99 [0.33,11.9]	1.16 [0.83,1.63]
**Panel C**	**Adjusted odds ratios for outcomes by admission to SU**^**3**^				
Mortality (30 days)	0.02 [0.00,0.18]	2.85 [0.60,13.53]	1.09 [0.17,7.02]	76.9 [0.91,64754]	0.85 [0.43,1.67]
Mortality (90 days)	0.20 [0.04,0.94]	4.76 [1.09,20.71]	0.85 [0.20,3.66]	4.50 [0.14,147]	1.45 [0.82,2.55]
Mortality (365 days)	0.40 [0.14,1.18]	1.83 [0.51,6.57]	0.84 [0.31,2.32]	1.04 [0.11,9.88]	1.12 [0.72,1.74]
**Panel D**	**Adjusted odds ratios for admission to SU by GCS/NIHSS**^**4**^				
GCS	1.17 [1.06,1.28]	0.87 [0.79,0.95]	1.07 [0.87,1.32]	0.89 [0.75,1.06]	0.93 [0.89,0.98]
NIHSS	0.93 [0.88,0.98]	1.22 [1.05,1.42]	0.90 [0.83,0.97]	1.09 [1.00,1.89]	1.04 [1.01,1.07]

Note 1: Odds Ratio [95% C.I.] from logit estimates. C.I. based on robust standard errors.

Note 2: ORs from logit of Outcome on Admission to SU (controlling for GCS and other variables).

Note 3: ORs from logit of Outcome on Admission to SU (controlling for GCS, NIHSS and other variables).

Note 4: ORs from logit of Admission to SU by GCS and NIHSS (controlling for other variables).

### Analysis - propensity score stratification and weighting

We checked for the imbalance in the values of the observed characteristics for patients admitted to SU and GMW using univariate measures of differences in means. Large and significant differences were observed in many covariates. Hence we re-estimated the logits for admission to SU or GMW but also included interactions of variables that were identified in the previous univariate analysis to be significant. The predicted values from the logit, which is the probability that a patient will be admitted to SU, was then used as the propensity score for the patient in a given center. Patients admitted to SU were assigned a weight of the reciprocal of the predicted probability of admission to SU while those admitted to GMW were assigned a weight of the reciprocal of the probability of admission to GMW. Weighted differences in means between SU and GMW were then computed to test if the propensity scores achieved reasonable balance among observables between SU and GMW [2,4,8,9]. Weighted and un-weighted differences in the covariates are summarized in Tables 3.

**Table 3:** Difference in case mix and patient characteristics by SU and GMW

	**London**		**Kaunas**		**Dijon**		**Florence**	
	**Pre**	**Post**	**Pre**	**Post**	**Pre**	**Post**	**Pre**	**Post**
GCS	2.014 ^*a*^	-0.157	-2.531 ^*a*^	-0.416	0.225	-0.086	-0.432	-0.057
	0.000	0.731	0.000	0.150	0.217	0.770	0.527	0.938
Swallow test	-0.120 ^*c*^	-0.035	0.123 ^*a*^	0.008	-0.185 ^*a*^	-0.017	-0.071	-0.036
(1/0, 1 if fail)	0.058	0.668	0.007	0.913	0.000	0.765	0.458	0.701
Age	0.192	0.800	9.912 ^*a*^	3.327 ^*b*^	-4.483 ^*a*^	-0.796	-4.720 ^*c*^	-0.676
	0.917	0.714	0.000	0.050	0.003	0.612	0.063	0.769
Age square	-66.163	131.689	1,398.868 ^*a*^	406.985 ^*c*^	-579.525 ^*a*^	-102.443	-616.334 ^*c*^	-89.409
	0.785	0.646	0.000	0.100	0.004	0.635	0.078	0.782
Male	0.041	-0.038	-0.117 ^*b*^	0.016	0.002	-0.003	0.073	-0.014
(1/0: 1 if true)	0.527	0.624	0.050	0.868	0.970	0.958	0.504	0.902
Age*Male	3.959	-1.331	-4.488	2.435	-1.957	-0.791	3.068	-1.349
	0.389	0.791	0.279	0.699	0.636	0.864	0.698	0.871
Stroke type: CI	0.130 ^*a*^	-0.021	-0.006	-0.009	-0.017	0.009	0.045	-0.010
(1/0: 1 if true)	0.006	0.659	0.866	0.847	0.549	0.742	0.563	0.911
Prestroke - AF	-0.038	-0.005	0.168 ^*a*^	0.077	0.063	0.015	-0.065	-0.027
(1/0: 1 if true)	0.436	0.933	0.001	0.384	0.178	0.784	0.452	0.755
Prestroke - DM	-0.045	-0.071	0.004	0.016	-0.006	-0.003	0.009	-0.004
(1/0: 1 if true)	0.416	0.355	0.911	0.821	0.892	0.944	0.922	0.964
Prestroke - HT	0.127 ^*b*^	-0.049	-0.039	0.047	-0.044	-0.000	-0.105	-0.008
(1/0: 1 if true)	0.047	0.492	0.491	0.496	0.414	0.996	0.327	0.939
Prestroke - MI	-0.010	-0.002	0.008	0.040	-0.049	-0.022	-0.062	-0.029
(1/0: 1 if true)	0.782	0.959	0.854	0.613	0.189	0.556	0.441	0.709
Needed help last 2wks	-0.079	0.412	0.226 ^*a*^	0.711	-0.145 ^*a*^	-0.132 ^*a*^	-0.081	-0.014
(1/0: 1 if true)	0.123	0.318	0.000	0.176	0.001	0.001	0.355	0.888
Able to walk	0.043	-0.067			0.203 ^*a*^	0.115 ^*c*^		
(1/0: 1 if true)	0.509	0.407			0.000	0.072		
Lift both arms	0.108 ^*c*^	0.002	-0.463 ^*a*^	-0.449 ^*a*^	0.079	-0.016		
(1/0: 1 if true)	0.099	0.980	0.000	0.000	0.147	0.799		
Home alone	-0.000	0.014	0.109 ^*b*^	0.071	-0.069	0.000		
(1/0: 1 if true)	1.000	0.855	0.018	0.323	0.192	0.995		
Home w./ others	0.065	-0.040	-0.211 ^*a*^	-0.075	0.054	0.014		
(1/0: 1 if true)	0.320	0.616	0.000	0.315	0.336	0.820		
Supportive living	-0.086 ^*a*^	0.002			-0.019	-0.006		
(1/0: 1 if true)	0.004	0.937			0.474	0.819		
LT hospital care	0.010	0.009			-0.059 ^*b*^	-0.009		
(1/0: 1 if true)	0.593	0.569			0.017	0.805		
Home unknown	0.010	0.015	0.103 ^*a*^	0.004	0.093 ^*a*^	0.001		
(1/0: 1 if true)	0.593	0.112	0.000	0.827	0.008	0.971		

Difference and p-value: Table provides difference (SU-GMW) in covariates (p-value listed below the difference).

Pre: Difference and p-value before adjusting by propensity score.

Post: Difference and p-value after applying weights based on propensity score.

Significance: Superscripts a, b, c indicate if p-value is less that.01,.05 and.10 respectively.

Abbreviations: Stroke Unit (SU); General Medical Ward (GMW); Glasgow Coma Scale (GCS); Cerebral Infarction (CI); Atrial Fibrillation (AF); Diabetes Mellitus (DM); Hypertension (HT); Myocardial Infarction (MI); Long Term (LT).

Based on the propensity scores, two methods were used to assess the impact of SU on outcomes. In the first method, using observations on the common support, we stratified the observations based on the propensity score. In the case of London and Kaunas, five strata were defined (quintiles). For Dijon and Florence, observations were divided into three strata. Dijon has a very low mortality rate and hence division into five strata leads to zero mortality in some of the subgroups, thus making computation of odds ratio for the strata in question infeasible. Similarly, there are relatively few observations available for Florence (93 total) again making five strata infeasible. Hence for these two centers, only three strata were used. Nonetheless, in each case, whether three or five strata were used, the first two moments of the propensity score between SU and GMW observations were compared within each strata to further check for adequacy of balance. Odds ratio of mortality for admission to SU were computed within each strata and a weighted average across strata was derived based on weights proportional to the inverse of the variance of the estimates [15]. In the second method, we computed the odds ratios based solely on the weights assigned to observations (reciprocal of probability for SU and reciprocal of one minus the probability for GMW) rather than stratification by propensity scores. This method was used to test for the robustness of the results implied by the stratification method. The results from the two methods (stratification or weighting) are given in Table 4 and are listed as PSM1 and PSM2 respectively.

**Table 4:** Odds ratios and confidence intervals – propensity score adjusted

**Outcome**	**London**	**Kaunas**	**Dijon**	**Florence**	**Combined**
	**Odds ratios for outcomes by admission to SU – PSM method 1**				
Mortality (30 days)	0.49 [0.25,0.99]	1.77 [0.98,3.19]	1.81 [0.42,7.88]	1.51 [0.39,5.82]	1.09 [0.78,1.51]
Mortality (90 days)	0.66 [0.34,1.29]	2.29 [1.29,4.07]	0.72 [0.25,2.08]	1.52 [0.48,4.81]	1.18 [0.88,1.58]
Mortality (365 days)	0.72 [0.39,1.35]	2.75 [1.50,5.04]	0.70 [0.33,1.46]	1.41 [0.49,4.10]	1.15 [0.88,1.49]
	**Odds ratios for outcomes by admission to SU – PSM method 2**				
Mortality (30 days)	0.55 [0.26,1.16]	1.37 [0.66,2.84]	2.11 [0.48,9.25]	1.24 [0.35,4.46]	0.94 [0.68,1.31]
Mortality (90 days)	0.75 [0.37,1.52]	2.42 [1.13,5.21]	0.82 [0.27,2.52]	1.28 [0.41,3.96]	1.06 [0.79,1.41]
Mortality (365 days)	0.74 [0.38,1.47]	2.16 [1.02,4.59]	0.76 [0.36,1.63]	1.23 [0.42,3.61]	1.06 [0.82,1.37]

## Results and discussion

### Descriptive statistics

Relevant summary statistics are provided in Table 1. Of the 1,492 patients, 13% died within 30 days. The overall rate was highest in London (17%) and lowest in Dijon (5%). Across the four centers, 534 patients were admitted to SU wards of which 15% died within one month, while 958 patients were admitted to GMW wards, of which 12% died within one month. This higher 30 day mortality rate in SU is driven mostly by one center, Kaunas, where 44% of patients died in the SU while only 11% died in GMW. The mortality rate in Florence in SU is also higher than in GMW, but the difference is not statistically significant. In the other two centers (London and Dijon), the 30 day mortality is always lower in SU though again the difference is not statistically significant in the case of Djon. A similar pattern exists for the other two outcome measures: compared to GMW, mortality rates are lower in SU in London and Dijon, but higher in SU in Kaunas and Florence and not significantly different across SU or GMW in Dijon and Florence.

Overall 35.7% of the patients were admitted to SU (534 out of 1492). However, there is a significant imbalance across centers. Admission to SU in Dijon is 35.5% and in Florence it is 39.4%, but in London 80% of the patients are admitted to SU while in Kaunas only 11% are admitted to SU. Similarly, the mean value of GCS for patients admitted to SU versus those admitted to GMW in both Dijon and Florence are very close to each other (for example in Florence the average values are 13.15 and 13.58 respectively) but there is a large difference in the mean values of GCS in both London and Kaunas. In London, the mean GCS value of patients admitted to SU is 2 points higher than those in GWM (13.19 vs. 11.18) while for Kaunas the mean value of GCS for patients in SU is 2.5 points lower than in GMW (11.47 vs 14).

### Multivariate logit analysis

Panel A of Table 2 provides the unadjusted odds ratios (OR) of mortality by admission to SU versus GMW. For instance, the OR for 30 day mortality in London is 0.25 for patients admitted to SU relative to patients admitted to GMW and the OR is statistically significantly different from one. For each of the three outcomes, the unadjusted odds ratios are significantly lower than one for London, higher than one and significant for Kaunas, and lower than one and higher than one for Dijon and Florence respectively, but not statistically different from one for the latter two cities. The last column provides ORs when the observations from the four centers are combined. The ORs for the three outcomes are above one because the combined data is dominated by large number of observations from Kaunas where the overall mortality rates in SU are much higher than in any other center.

Mortality odds ratios by admission to SU or GMW, adjusted by severity of stroke as measured by GCS, patient demographics and other case-mix variables listed earlier are given in Panel B of Table 2. After controlling for these factors, the mortality OR by admission to SU was significantly lower than one for the 30 day mortality in London but not significantly different from one for the 90 day or one year mortality. For Kaunas, it continued to be above one but was significant for only the one year mortality. For Dijon and Florence, the 95% C.I. always included one in the interval. Since the GCS can suffer from ceiling effect, we repeated the analysis above using NIHSS as a measure of severity of stroke. This reduced the working sample by about 25% but other than the point differences in OR, it resulted in qualitatively similar findings and are listed in Panel C of Table 2. The mortality OR for admission to SU is significantly lower than one for the 30 and 90 day mortality in London, higher than one in Kaunas (significant for the 90 day mortality), and as before, not significantly different from one for the three outcome measures in Dijon and Florence. When we combine observations across the four centers, adjusted OR by admission to SU or GMW are fairly similar in Panel B and Panel C. In each case the combined OR is not significantly different from one, but this is to be expected since, (1) it ignores the differences in these centers and (2) because the lower than one OR for London is combined with the higher than one OR in Kaunas, such that the average effect is much closer to one.

Based on these adjusted ORs by center, a tentative conclusion is that patients admitted to SU in London have better survival probabilities than their counterparts admitted to GMW, at least in the short and intermediate term (30 and 90 days). Similarly, patients admitted to SU in Kaunas tend to fare much worse when admitted to SU, at least in the intermediate and the long run (90 and one year mortality) while patients in Dijon and Florence are at par in terms of survival in SU and GMW.

However, these results are driven in part by the type of patient admitted to SU or GMW. The bottom part of Table 2 (Panel D) provides the adjusted OR for admission to SU by the initial severity of stroke as measured by Glasgow Coma Scale (GCS) or the National Institutes of Health Stroke Scale (NIHSS). The GCS ranges from 3-15 and a higher value of GCS indicates a less severe stroke whereas the NIHSS ranges from 0-42, with higher values reflecting more severe case. Controlling for other factors, a one point increase in GCS is associated with a 17% increase in odds of admission to SU in London, a 13% decrease in odds of admission to SU in Kaunas, a 7% increase in odds of admission to SU in Dijon, and an 11% decrease in odds of admission to SU in Florence. Of these, the OR for London and Kaunas are significant while Dijon and Florence are not. Patients with less severe stroke are more likely to be admitted to SU in London while those with more severe stroke are more likely to be admitted to SU in Kaunas and there is no statistical evidence at conventional significance levels to suggest that initial severity of stroke plays a significant role in admission to SU or GMW in Dijon and Florence (though for Florence, which had the least number of observations, the p-value is 0.16). Repeating the analysis with NIHSS shows a similar selection process. A one point increase in NIHSS value decreases the odds of admission to SU in London by 7%, increases the odds of admission by 22% in Kaunas, decreases it by 10% in Dijon and increases it by 9% in Florence and the OR is significantly different from one even for Dijon.

### Propensity score analysis

Patients admitted to SU and GMW may differ in other characteristics as well. Table 3 lists the differences in means, along with the associated p-values, for the difference for each of the covariates by SU and GMW (under the columns labeled 'Pre’). In London, on average, patients admitted to SU score two points higher on GCS score and are 12% less likely to fail the swallow test at admission. Some differences also exist on the type of stroke and pre-existing co-morbidity of hypertension. By contrast, patients admitted to SU in Kaunas are very different. On average, the SU patients score two and half points lower on the GCS score, are 12% more likely to fail the swallow test, SU patients are 9.9 years older and about 16.8% also suffer from atrial fibrillation, a known risk factor for stroke. In Dijon and Florence there is no significant difference in the GCS score among patients admitted to SU or GMW. However, those admitted to SU in Dijon are 18.5% less likely to fail the initial swallow test and are almost four and a half years younger, while in Florence only age appears to be significant and only at the 10% significance level.

To adjust for these differences in observables, a propensity score for each patient was computed as the logit predicted probability of admission to SU. All the original covariates, along with the interactions among the covariates that were initially significantly different were used to predict the probability of admission to SU. Weighting observations based on the propensity score, the differences in covariates were no longer significant and are given in the columns labeled 'Post’ in Table 3. The results show that a good balance was achieved so that outcomes can be analyzed conditional on this score.

The odds ratios based on the two propensity score methods described earlier are given in Table 4. Two things are worth pointing out about odds ratios listed in this table. First, compared to the odds ratios listed earlier using multivariate logits, the odds ratios for London have increased in magnitude and are closer to one while for Kaunas they have decreased in magnitude and are again closer to one. For Florence, they have also decreased (and are closer to one) and only for Dijon, they have increased slightly from there original estimates and are further away from one. These changes in the magnitude of the odds ratios, particularly in the direction they have moved, show that the traditional multivariate logit methods do not adequately account for the differences in patient characteristics/case-mix when the underlying distributions are very different across the patients admitted to SU and GMW. Second, except for the odds ratio for 90 and 365 days mortality in Kaunas, none of the other point estimates are significantly different from one.

It is also worth comparing these ORs with those from randomized control trials. The most recent Cochrane review (a meta analysis) compares death by the end of scheduled follow-up (typically a year) for patients admitted to comprehensive stroke wards versus general medical ward from 11 randomized control trials [16], [see pp. 75]. The ORs in these RCTs range from as low as 0.11 to as high as 1.50 with the mean value of 0.77 (95% CI 0.63 to 0.93). While there is large variation in these RCT results, to the extent that the average can be used as a 'benchmark’, the ORs from the two propensity scores methods for 365 days mortality for London (0.72 or 0.74) and Dijon (0.70 or 0.76) compare well with this RCT average. Even the OR for Florence, with its large confidence interval due to the small sample size, includes the RCT average. Only Kaunas, which admits significantly sicker patients to SU seems to be an outlier. By comparison, the OR for 356 days for London from the earlier multivariate logit method (0.55 or 0.40, see panel B or C from Table 2) is much below the RCT average of 0.77 but the ORs from Dijon (0.80 or 0.84) stay close to both the PSM values and the RCT average. Recall however that London exhibited a selection bias (less severe stroke patients admitted to SU) while Dijon does not show similar selection bias. To the extent that the multivariate logits and the PSM used the same control variables, this comparison with RCTs also shows that in observational studies PSM methods can better account for overt selection.

### Robustness

We re-estimated the models using alternative functional forms or measures of stroke severity. The results were robust to other specifications such as the inclusion of quadratic and cubic terms of the GCS score, switching to categorical classification for the GCS as low < 9, medium 10–12, and high > 13, or alternatively to use individual components of GCS for eye opening score, motor response score, and verbal response score. Other changes in the specifications included replacing GCS with the Barthel score post initial stroke, omitting the swallowing test variable, dropping additional control variables (hypertension, myocardial infraction and medication therapy), and including observations on patients with subarachnoid haemorrhage in the analysis. In each of the cases above, the point estimates for the earlier reported odds ratios were only slightly different, but in none of the cases did the results change in any substantial way.

### Limitations

The odds ratios reported in this paper should not be taken at face value for at least two reasons. First, propensity score methods are best applicable in large data sets. In the current analysis, the total sample is sub grouped by cities to account for the underlying differences in care between cities. This leads to relatively small sub samples, especially for Florence, and hence the large confidence intervals for odds ratios could also be attributed to a small sample problems (albeit the same small samples are used in both methods). Second, while the propensity score method accounts for overt selection, the underlying assumption is of strongly ignorable treatment assignment given the observed covariates. The assumption implies that no systematic unobserved pretreatment differences exist between the two groups that are related to the outcome measure. Nonetheless, patients who appear similar in terms of observed covariates may differ in other important dimensions not observed in the data. Thus, if in addition there is a hidden (or covert) bias, neither the multivariate regressions nor any of the propensity score methods can produce unbiased estimates of relative effectiveness of admission to stroke unit. To do so would require finding and using an instrumental variable that predicts reasonably well which patient is admitted to SU or GMW, but at the same time cannot predict the probability of death or poor outcome for the patient. Such a variable is not available in the EROS database but future work using other databases may be able to overcome this difficulty.

## Conclusions

Benefits of organized stroke care have been debated for the past three decades [17-19]. Randomized control trials, as well as meta analysis of randomized trials have typically shown benefits to patients admitted to stroke units over general wards and have formed the foundation of evidence based stroke care since the mid 1990’s [16,20,21]. Since controlled trials are often conducted under special conditions and exclude certain populations, the general findings that patients in stroke unit are more likely to survive, return home and regain independence are subject to criticism that these special conditions do not apply in real settings [22,23]. As a response, researchers have also compared outcomes between stroke units and other wards in observational studies and reported mixed findings [22,24,25]. Gompertz et al. [22] found that outcomes did not differ across patients admitted to adjacent districts in London where one had no special stroke services while the other had comprehensive stroke services. Using data from Swedish National Registry, Stegmayar et al. [24] report that stroke care was effective while Terent et al. [25], who also use data from Sweden, find that the magnitude of benefits from admission to stroke unit is smaller than that reported in randomized trials. They also report that there was no difference in outcomes between stroke unit and other wards if the patient had impaired consciousness at admission. Nonetheless, a recent meta analysis of observational studies [23] concluded that the observed benefits of admission to stroke unit are comparable to those reported in clinical trials but as was noted in the review as well as elsewhere [26], there were a number of limitations in the study including, importantly, possible selective admission to stroke units and incomplete case-mix adjustments.

The foregoing analysis shows that traditional multivariate regression based methods tend to overestimate the impact of admission to stroke unit on outcomes in the presence of overt selection. When there is favorable selection of patients, e.g. London, the odds ratios are downward biased. When there is unfavorable selection, e.g., Kaunas, the odds ratios are upward biased. In both London and Kaunas, where selection is most severe, odds ratios based on multivariate logits are significantly below or above one. Correcting explicitly for this imbalance in covariate distribution, especially for the severity of stroke, leads to a much more modest impact of stroke unit on patient mortality. In the case of London, there is no statistical difference in mortality between SU and GMW. In the case of Kaunas, the mortality rates are higher in SU and the intermediate and long term (90 and 365 days) odds ratio stay significantly above one, though the magnitude decreases once selection of more severe and older patients in SU is accounted for via propensity scores. In the case of the other two cities, Dijon and Florence, where the distribution of severity is more balanced, the conclusions from the two methods, multivariate regressions and propensity score methods do not change, i.e., admission to stroke unit does not lead to statistically different outcomes as the confidence interval for the odds ratios always include one.

Our analysis does not support the hypothesis that admission to SU leads to lower mortality compared to patients admitted to GMW. The lower odds of death in SU in London are driven by selection of patients with lower initial severity of stroke while the higher odds of death in SU in Kaunas are driven, in part, by selection of patients with greater initial severity of stroke. The other two centers exhibit neither any significant selection by severity of stroke, nor do they, consequently, show an difference in outcomes by SU or GMW wards.

The different estimates of odds ratios presented in this paper highlight that alternative conclusions about the relative effectiveness of stroke units can be drawn depending on if overt selection is accounted for or not in the estimation methods. Moreover, these estimates show that the case for cost-effectiveness of stroke units, compared to care in other wards is not a closed case (even the earlier cited RCTs show ORs ranging from.11 to 1.50). For instance, Patel et al. compared outcomes and costs from a randomized control trial where patients either received care in a stroke unit, in the care of a stroke team (in other wards) or in domiciliary care [27]. While the percentage of patients who avoided death or institualization at one year was 87% in stroke unit, 69% in stroke team and 78% in domiciliary care respectively, the stroke care unit was most expensive while domiciliary care the cheapest. At a willingness to pay of £30,000 per additional QALY, the probability that the stroke unit or stroke team are the most optimal of the three strategies was only 29% and the incremental cost effectiveness ratio (ICER) between stroke unit and domiciliary care ranged from £67K to £137K (depending on the cost perspective used). Similarly, the reported ICER of £10,661 of SU compared to general medical ward in Saka et al. [28] is based on cost estimates from observational data from South London but effectiveness measures from an earlier randomized trial [29] of conventional care compared with an early discharge policy at two hospitals in the same area. In either of these studies, the ICER figures are likely to be much higher if the incremental effectiveness in these ratios is replaced with figures computed from observational studies that account for the selection bias.

## Competing interests

The authors declare that they have no competing interests.

## Authors’ contributions

CW is the coordinator of the study and provided feedback on drafts and results and is responsible for data collection and data integrity. AR, PL and MD provided background information based on their experience as experts and investigators in this field and commented on drafts. FB and IW wrote the initial draft and FB provided the data analysis. All authors read and approved the final manuscript.
